# Agreement between venous blood gas and serum biochemistry measurements for sodium, potassium, chloride, and glucose in children with acute gastroenteritis: A method-comparison study

**DOI:** 10.1097/MD.0000000000049763

**Published:** 2026-07-17

**Authors:** Özlem Erdede, Dilara Lahut, Erdal Sari, Rabia Gönül Sezer Yamanel

**Affiliations:** a Department of Pediatrics, Zeynep Kamil Maternity and Children’s Disease Training and Research Hospital, University of Health Sciences, Istanbul, Beykoz, Turkey.

**Keywords:** acute gastroenteritis, electrolyte agreement, method comparison, pediatric, serum biochemistry, venous blood gas analysis

## Abstract

Acute gastroenteritis (AGE) is a common cause of pediatric emergency department (ED) visits and is frequently associated with dehydration and metabolic disturbances. Rapid assessment of electrolytes and glucose is essential in clinical management. Although venous blood gas analysis provides faster results, its agreement with standard serum biochemistry in pediatric AGE remains unclear. This retrospective observational study included children aged 1 month to 18 years who presented to the pediatric ED with AGE between January 1, 2024, and December 31, 2025, and underwent paired venous BGA and serum biochemical testing during the same visit. A total of 1853 paired measurements were obtained from 1191 patients, as some children contributed multiple paired measurements during repeated testing. Method comparison was performed according to Clinical and Laboratory Standards Institute (CLSI) EP09-A3 guidelines using intraclass correlation coefficients (ICC), paired comparisons, Pearson correlation, and Bland–Altman analysis. Both analyzers demonstrated acceptable intra-device reliability (ICC > 0.70). However, agreement between venous blood gas and serum biochemistry was poor for sodium, potassium, chloride, and glucose, with all inter-method ICC values below 0.70. Mean values differed significantly between methods for all parameters (*P* < .01*).* Bland–Altman analysis demonstrated wide limits of agreement (LOA) between the 2 methods; for example, glucose measurements showed a mean difference of 2.1 mg/dL with LOA ranging from −64.3 to 68.5 mg/dL. Venous blood gas electrolyte and glucose measurements are not interchangeable with serum biochemistry in children with acute gastroenteritis. While blood gas analysis may be useful for rapid screening or trend monitoring in urgent settings, confirmatory serum biochemical testing remains necessary for clinical decision-making in pediatric AGE.

## 1. Introduction

Acute gastroenteritis (AGE) is one of the leading causes of pediatric emergency department (ED) admissions worldwide and contributes substantially to dehydration, electrolyte disturbances, and hospitalization in children.^[1–3]^ Dehydration resulting from vomiting and diarrhea is the most common complication and is frequently associated with abnormalities in electrolytes – particularly sodium, potassium, and chloride – as well as glucose levels.^[4–6]^ In the ED setting, rapid assessment of electrolyte and glucose abnormalities is essential for appropriate clinical management.^[7]^

Although serum biochemistry testing is considered the reference standard for metabolic evaluation, turnaround times may be prolonged due to laboratory processing delays, particularly in high-volume EDs. Advances in technology have enabled modern blood gas analyzers to measure electrolytes and glucose using ion-selective electrodes, allowing for more rapid availability of results. Several studies have compared venous blood gas analysis–derived electrolyte and glucose measurements with standard laboratory biochemistry, reporting inconsistent and heterogeneous findings. While some studies have demonstrated acceptable agreement for selected analytes,^[8,9]^ others have identified clinically significant discrepancies that limit the interchangeability of these methods.^[10,11]^

Despite the increasing use of venous blood gas analysis in pediatric EDs, its reliability as a substitute for serum biochemistry remains uncertain in specific clinical scenarios. Previous studies have largely focused on heterogeneous pediatric populations or critically ill children rather than disease-specific cohorts. In contrast, our study specifically evaluates children presenting with acute gastroenteritis, a condition characterized by rapid fluid loss, dehydration, and acid–base disturbances that may influence agreement between whole-blood blood gas and serum biochemical measurements.

AGE is characterized by rapid fluid loss, dehydration, and frequent acid–base disturbances, which may further accentuate differences between whole-blood and serum-based measurement methods. Consequently, evidence addressing the agreement between venous blood gas and serum biochemical measurements in children with acute gastroenteritis remains limited.

Therefore, the present study aimed to evaluate the agreement between venous blood gas and serum biochemical measurements of sodium, potassium, chloride, and glucose in pediatric patients with acute gastroenteritis, and to assess whether venous blood gas analysis may serve as a rapid alternative to serum biochemistry in the pediatric emergency setting.

## 2. Materials and methods

### 2.1. Study design and setting

This retrospective observational study was conducted in the Pediatric ED of Zeynep Kamil Maternity and Children’s Training and Research Hospital, a tertiary-care referral center. The study period extended from January 1, 2024, to December 31, 2025.

### 2.2. Study population

Children aged 1 month to 18 years who presented to the pediatric ED with a diagnosis of AGE and underwent both venous blood gas analysis and routine serum biochemical testing during the same ED visit were eligible for inclusion.

The diagnosis of acute gastroenteritis was established based on clinical evaluation by the attending pediatric emergency physician and was defined as the acute onset of diarrhea (≥3 loose or watery stools within a 24-hour period), with or without vomiting, a symptom duration of <14 days, and the absence of an alternative explanatory diagnosis.

A total of 1191 individual patients met the inclusion criteria. During the study period, some patients presented to the ED on more than one occasion for separate episodes of acute gastroenteritis. In addition, during a single ED visit, repeat laboratory testing was occasionally performed at the discretion of the treating physician for clinical monitoring or result verification. Consequently, the final dataset comprised 1853 paired venous blood gas and serum biochemical measurements, derived from multiple ED visits and, in some cases, repeated measurements obtained during the same visit. Each paired measurement represented venous blood gas and serum biochemical samples obtained simultaneously during a single sampling event.

Patients with missing laboratory data, hemolyzed samples, incomplete medical records, or samples deemed unsuitable for analysis because of preanalytical or analytical issues (including hemolysis, insufficient sample volume, clot formation, prolonged processing time, or analyzer-reported errors) were excluded. Only valid paired measurements obtained during the same ED visit were included, in accordance with Clinical and Laboratory Standards Institute (CLSI) EP9-A3 guidelines.^[12]^

### 2.3. Data collection

Demographic characteristics (age and sex), date of admission, and laboratory results were retrospectively extracted from the hospital’s electronic medical record system. Venous blood gas and serum biochemical results were individually reviewed and paired using unique laboratory protocol numbers to ensure that measurements originated from the same patient encounter and the same sampling time.

## 3. Laboratory measurements

### 3.1. Biochemical analysis

Routine serum biochemical analyses were performed using the Roche Hitachi Cobas C6000 analyzer (Roche Diagnostics, Mannheim, Germany), which has been in clinical service at the institution since 2021. The analyzer was operated in accordance with the manufacturer’s instructions, and regular maintenance, calibration, and internal and external quality control procedures were conducted at manufacturer-recommended intervals, as documented in the laboratory device management system.

### 3.2. Blood gas analysis

Venous blood gas measurements were obtained using the Siemens RapidPoint 500 E blood gas analyzer (Siemens Healthineers, Germany), which has been in service since 2022. Blood gas samples were collected in heparinized syringes and analyzed immediately after collection. The device underwent routine internal and external quality control procedures and scheduled maintenance in accordance with the manufacturer’s recommendations and the institutional laboratory quality assurance program.

### 3.3. Measurement reliability

To assess intra-device measurement reliability, a subgroup of 270 patients with 3 repeated measurements obtained from the same blood sample was retrospectively identified from the laboratory database. Because the study was retrospective, these repeated analyses were not performed according to a predefined research protocol but occurred during routine clinical practice for result verification, confirmation of unexpected or critical values, or analyzer flags. All measurements were performed using the same analyzer under standardized laboratory conditions according to the institutional laboratory protocol.

Intraclass correlation coefficients (ICC) were calculated for each analyzer and each parameter, by using the model consistency with values >0.70 considered indicative of acceptable reliability.^[13]^ ICC values were calculated using a two-way mixed-effects model with consistency and 95% confidence interval.

### 3.4. Study parameters

The parameters evaluated for agreement between venous blood gas analyzer and serum biochemical analyzer measurements included: Sodium (Na), Potassium (K), Chloride (Cl), and Glucose.

### 3.5. Method comparison and statistical analysis

Method comparison and bias estimation between venous blood gas and serum biochemical measurements were performed in accordance with the CLSI EP9-A3 guideline, using paired patient samples. Because the primary aim of the study was to evaluate analytical agreement between measurement methods rather than patient-level clinical outcomes, each paired blood gas and serum biochemical measurement was treated as an independent observation in the statistical analyses.

Continuous variables were summarized as mean ± standard deviation or median (interquartile range), depending on data distribution. The distribution of the variables was assessed using the Shapiro–Wilk normality test before performing the statistical analyses. The relationship between measurement methods was assessed using Pearson correlation analysis. Agreement between the 2 measurement methods was assessed using Bland–Altman analysis. For each paired measurement, the difference between the 2 methods was plotted against their mean value. The mean difference (bias) and the limits of agreement (LOA) were calculated as the bias ± 1.96 times the standard deviation of the differences, representing the range within which 95% of the differences between the 2 methods are expected to fall. When interpreting the Bland–Altman results, the magnitude of the LOA was evaluated in relation to commonly accepted laboratory performance specifications. According to the Clinical Laboratory Improvement Amendments (CLIA) allowable total error limits, differences of approximately ± 4 mmol/L for sodium, ±0.5 mmol/L for potassium, ±5 mmol/L for chloride, and ±10% for glucose are generally considered clinically acceptable when comparing analytical methods.^[14]^ Interchangeability between methods was interpreted based on agreement rather than correlation alone. All paired measurements were treated as independent observations in accordance with the analytical aim of the study.

A *P*-value < .05 was considered statistically significant.

### 3.6. Ethical considerations

The study was conducted in accordance with the principles of the Declaration of Helsinki. Owing to the retrospective design and the use of anonymized data, informed consent was waived. Ethical approval was obtained from the local institutional ethics committee (Date: December 10, 2025; Approval No: 159). Due to the retrospective nature of the study and the use of anonymized patient data, the requirement for informed consent was waived by the institutional ethics committee.

## 4. Results

A total of 1853 paired venous blood gas and serum biochemical analyses obtained from 1191 pediatric patients presenting with acute gastroenteritis were included in the final analysis. The demographic characteristics of the study population are summarized in Table 1. The median age of the patients was 32 months (range: 1–256 months). Of the 1191 patients, 674 (56.59%) were male and 517 (43.41%) were female.

**Table 1:** Demographic characteristics of the study population (n = 1191).

Parameter	Value
Age (mo)	
Mean ± SD	51.46 ± 51.29
Median	32
Minimum–maximum	1–256
Sex, n (%)	
Male	674 (56.59%)
Female	517 (43.41%)

SD = standard deviation.

### 4.1. Intra-device reliability

Intra-device measurement reliability was assessed using 3 consecutive measurements obtained from blood samples of 270 patients. All ICC values for both the biochemistry analyzer and the blood gas analyzer exceeded the predefined threshold of 0.70, indicating good measurement reliability for all parameters evaluated. Detailed ICC values for sodium, potassium, chloride, and glucose are presented in Table 2.

**Table 2:** Intra-device reliability of the biochemistry analyzer and blood gas analyzer.

Parameter	Biochemistry analyzer ICC (95% CI)	Blood gas analyzer ICC (95% CI)
Na (mmol/L)	0.844 (0.723–0.956)	0.828 (0.793–0.949)
K (mmol/L)	0.812 (0.785–0.951)	0.832 (0.805–0.951)
Cl (mmol/L)	0.851 (0.831–0.965)	0.845 (0.823–0.961)
Glucose (mg/dL)	0.849 (0.829–0.933)	0.840 (0.817–0.965)

CI = confidence interval, Cl = chloride, glucose = blood glucose, ICC = intraclass correlation coefficient, K = potassium, Na = sodium.

### 4.2. Agreement between biochemistry analyzer and blood gas analyzer

The agreement between serum biochemistry analyzer (Measurement 1) and blood gas analyzer (Measurement 2) results was evaluated using intraclass correlation analysis. The ICC values were 0.518 (95% CI: 0.481–0.551) for sodium, 0.459 (95% CI: 0.417–0.466) for potassium, 0.517 (95% CI: 0.471–0.559) for chloride, and 0.508 (95% CI: 0.461–0.551) for glucose. All ICC values were below the predefined agreement threshold of 0.70, indicating poor agreement between the 2 measurement methods. Accordingly, serum biochemistry and blood gas analyzer measurements were considered not interchangeable (Table 3).

**Table 3:** Agreement between biochemistry analyzer and blood gas analyzer measurements.

Parameter	ICC (95% CI)
Na (mmol/L)	0.518 (0.481–0.551)
K (mmol/L)	0.459 (0.417–0.466)
Cl (mmol/L)	0.517 (0.471–0.559)
Glucose (mg/dL)	0.508 (0.461–0.551)

CI = confidence interval, Cl = chloride, glucose = blood glucose, ICC = intraclass correlation coefficient, K = potassium, Na = sodium.

### 4.3. Comparison of mean values

Mean electrolyte and glucose values obtained from the biochemistry analyzer and the blood gas analyzer are compared in Table 4. Mean sodium and chloride concentrations measured by the blood gas analyzer were significantly higher than those measured by the biochemistry analyzer (both *P* < .001). In contrast, mean potassium and glucose concentrations measured by the blood gas analyzer were significantly lower compared with serum biochemistry results (*P* < .001 and *P* = .009, respectively). Although statistically significant differences were observed for all parameters, the magnitude of the differences was relatively small for sodium, potassium, and chloride, while glucose showed greater variability, suggesting potentially higher clinical inconsistency.

**Table 4:** Comparison of mean electrolyte and glucose values between biochemistry analyzer and blood gas analyzer.

Parameter	Biochemistry (mean ± SD)	Blood gas (mean ± SD)	*P* value
Na (mmol/L)	136.7 ± 3.28	137.52 ± 3.29	<.001
K (mmol/L)	4.48 ± 0.52	4.17 ± 0.50	<.001
Cl (mmol/L)	102.63 ± 5.00	104.05 ± 4.88	<.001
Glucose (mg/dL)	98.77 ± 29.81	96.71 ± 29.21	.009

Cl = chloride, glucose = blood glucose, K = potassium, Na = sodium, SD = standard deviation.

### 4.4. Correlation analysis

Pearson correlation analysis demonstrated statistically significant positive correlations between biochemistry analyzer and blood gas analyzer measurements for sodium (*R* = 0.447), potassium (*R* = 0.262), chloride (*R* = 0.349), and glucose (*R* = 0.341) (all *P* < .001). However, the strength of these correlations ranged from weak to moderate (Table 5).

**Table 5:** Pearson correlation between biochemistry analyzer and blood gas analyzer measurements.

Parameter	*r*	*P* value
Na (mmol/L)	0.447	<.001
K (mmol/L)	0.262	<.001
Cl (mmol/L)	0.349	<.001
Glucose (mg/dL)	0.341	<.001

Cl = chloride, glucose = blood glucose, K = potassium, Na = sodium, r = Pearson correlation coefficient.

### 4.5. Bland–Altman analysis

**Figure 1.**
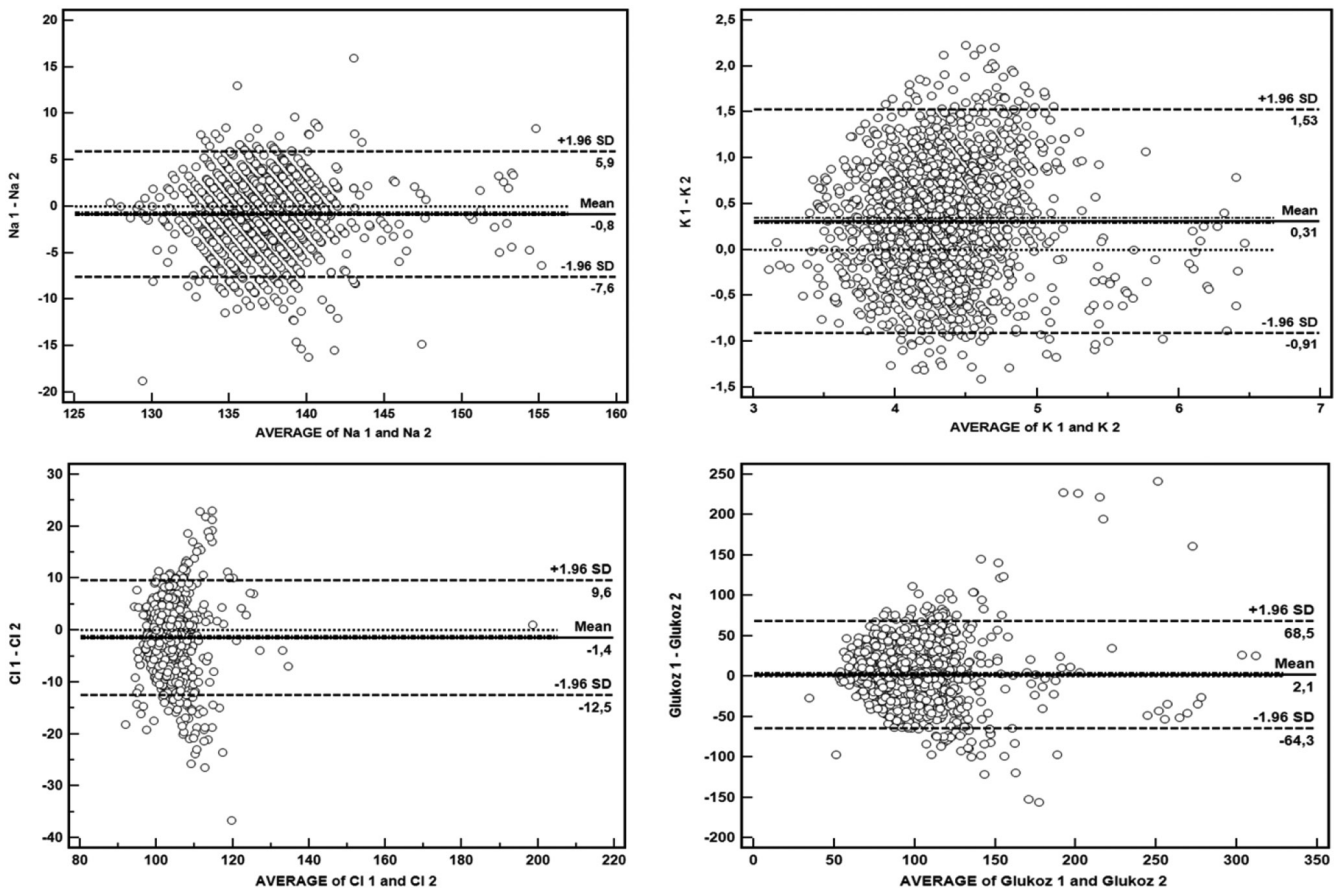
Bland–Altman plots illustrating the agreement between the biochemistry analyzer and the blood gas analyzer for (A) sodium (Na), (B) potassium (K), (C) chloride (Cl), and (D) glucose measurements. The solid horizontal line represents the mean difference (bias), and the dashed lines indicate the 95% limits of agreement (±1.96 SD). SD = standard deviation.

Bland–Altman analysis revealed a mean difference of −0.8 for sodium, with LOA ranging from −7.6 to 5.9. For potassium, the mean difference was 0.31, with LOA between −0.91 and 1.53. Chloride measurements showed a mean difference of −1.4, with LOA ranging from −12.5 to 9.6. Glucose measurements demonstrated a mean difference of 2.1 and very wide LOA from −64.3 to 68.5. The wide LOA observed for all parameters indicate substantial variability between the 2 measurement methods and further support the lack of interchangeability between serum biochemistry and blood gas analyzer measurements. Notably, glucose showed the widest LOA (−64.3–68.5), representing the greatest dispersion and the poorest agreement between the 2 methods among all parameters (Table 6, Fig. 1).

**Table 6:** Bland–Altman analysis of agreement between biochemistry analyzer and blood gas analyzer measurements.

Parameter	Mean difference	Limits of agreement
Na (mmol/L)	−0.8	−7.6 to 5.9
K (mmol/L)	0.31	−0.91 to 1.53
Cl (mmol/L)	−1.4	−12.5 to 9.6
Glucose (mg/dL)	2.1	−64.3 to 68.5

Cl = chloride, glucose = blood glucose, K = potassium, Na = sodium.

## 5. Discussion

In this large retrospective pediatric ED cohort of children with AGE, we demonstrated that venous blood gas analyzer measurements of sodium, potassium, chloride, and glucose are not interchangeable with central laboratory biochemistry results. Although intra-device reliability was high for both analyzers, inter-method agreement was consistently poor. Bland–Altman analyses revealed wide LOA for all analytes, particularly for glucose, indicating substantial variability between methods. In line with CLSI EP09-A3 principles, these findings emphasize that agreement, rather than correlation alone, should guide interchangeability decisions.^[12]^ When interpreting these findings, the magnitude of the LOA should be considered in relation to commonly accepted laboratory performance specifications rather than strict clinical decision thresholds. In this context, commonly used allowable error limits suggest that differences of approximately ±4 mmol/L for sodium, ±0.5 mmol/L for potassium, ±5 mmol/L for chloride, and ±10% for glucose may be considered acceptable when comparing analytical methods.

Our findings are consistent with previous pediatric and mixed-population studies reporting method-dependent differences between blood gas and laboratory measurements. In a pediatric single-center study, Konuksever et al reported that despite apparent correlations, method-dependent differences limited the interchangeability of blood gas and laboratory electrolyte measurements.^[10]^ Similarly, Yi et al demonstrated that blood gas analyzers may yield systematically different electrolyte and glucose values compared to chemistry analyzers, with variability depending on the specific analyte evaluated.^[15]^ Bardak et al further reported that sodium measurements obtained from venous blood gas analysis may be influenced by acid–base status, whereas potassium measurements appear more robust across pH conditions, underscoring the need for cautious interpretation of sodium results and laboratory confirmation with laboratory testing when clinical findings are inconsistent.^[11]^

In contrast, some studies have reported acceptable agreement for selected parameters. Pouryahya et al observed strong correlations for sodium and potassium in an adult ED population, suggesting potential clinical utility in that setting.^[8]^ Likewise, Kilic et al reported good agreement for sodium and potassium measurements in pediatric kidney transplant recipients.^[9]^ However, these findings should be interpreted with caution, as they were derived from different patient populations and clinical contexts. The distinct physiological characteristics of AGE may further amplify differences between measurement methods.

In our cohort, blood gas analyzer measurements were systematically higher for sodium and chloride and lower for potassium and glucose compared to serum biochemistry results, indicating a systematic bias between the 2 methods.

Several analytical and preanalytical factors may explain these differences. First, differences between direct and indirect ion-selective electrode methodologies and matrix effects related to whole blood versus serum or plasma can result in measurement discrepancies.^[16]^ Second, heparin-related dilution and sample handling effects are recognized sources of bias in blood gas analysis, particularly in pediatric settings with small sample volumes.^[17]^ Third, glucose measurements obtained from blood gas analyzers are sensitive to preanalytical conditions, which may contribute to variability between methods.^[18]^

Blood samples in our study were obtained simultaneously and analyzed at the same site, which may have reduced the impact of preanalytical delays; however, these factors were not directly measured.

This strengthens the interpretation that the discrepancies primarily reflect intrinsic method- and matrix-related differences rather than workflow-related delays. This is particularly relevant when interpreting the observed bias, as it supports that the differences between methods are likely analytical rather than procedural in origin.

Clinical guidelines for the management of AGE emphasize the importance of early assessment of dehydration and metabolic disturbances in children.^[1]^ Previous studies in pediatric AGE populations have shown that hypoglycemia and electrolyte abnormalities may occur and can be clinically relevant, particularly in younger or hospitalized children.^[18]^ However, most method-comparison studies between blood gas and laboratory measurements have not focused specifically on AGE, instead evaluating heterogeneous pediatric populations or different clinical conditions.^[19]^ Given the characteristic physiological changes in AGE, including rapid fluid loss and acid–base disturbances, these factors may amplify analytical differences between measurement methods. Therefore, our study addresses an important gap by evaluating method agreement specifically in a pediatric AGE cohort.

From a clinical perspective, although blood gas testing provides rapid results, our findings indicate that blood gas–derived electrolyte and glucose values should not replace serum biochemistry for clinical decision-making in pediatric AGE. Blood gas measurements may be useful as an initial screening tool or for trend monitoring in urgent settings; however, confirmatory serum testing remains necessary, particularly for values near clinical decision thresholds such as dysnatremia, potassium abnormalities, and suspected hypoglycemia.

The strengths of this study include the large, disease-specific pediatric AGE cohort, the use of paired measurements obtained during the same ED visit, and the application of multiple complementary statistical approaches aligned with CLSI EP09-A3 recommendations, including intraclass correlation and Bland–Altman analyses rather than reliance on correlation alone. This study also has several limitations. Due to its retrospective design, there is a potential for selection bias related to clinician-directed testing. In addition, outcome-based analyses were not performed; therefore, it was not possible to determine how often the observed measurement discordance would have resulted in changes in clinical management. However, given the magnitude of disagreement observed – particularly for glucose – such discrepancies may be clinically relevant, especially for values near treatment thresholds. Additionally, we did not stratify agreement by dehydration severity, acid–base status, or hematocrit. Data on dehydration severity and detailed clinical assessment parameters were not systematically recorded and, therefore, could not be included in subgroup analyses. Although acid–base parameters were available from blood gas analysis, they were not documented in a standardized manner suitable for stratified evaluation. Similarly, hematocrit values were only available for a subset of patients and were not uniformly obtained across the entire cohort. Additionally, since multiple measurements were obtained from some patients, the assumption of independence between observations may not be fully met. Treating repeated measurements as independent may introduce within-patient correlation, which could lead to underestimation of variability and affect the precision of agreement estimates.

## 6. Conclusion

In children presenting to the ED with acute gastroenteritis, venous blood gas measurements of sodium, potassium, chloride, and glucose showed poor agreement with serum biochemistry despite good intra-device reliability. These findings indicate that blood gas and laboratory measurements are not interchangeable in this setting. Although blood gas analysis provides rapid results, serum biochemistry remains necessary for accurate clinical decision-making, particularly for values near treatment thresholds. Notably, glucose demonstrated the widest LOA among all parameters, indicating the greatest variability between the 2 methods and warranting particular caution in clinical interpretation.

## Acknowledgments

Thanks for the statistician Rana Konyalioğlu for statistic analyze. (www.rkonyalioglu@gmail.com)

## Author contributions

**Conceptualization:** Özlem Erdede.

**Data curation:** Erdal Sari.

**Formal analysis:** Dilara Lahut.

**Investigation:** Erdal Sari.

**Methodology:** Özlem Erdede, Dilara Lahut.

**Project administration:** Rabia Gönül Sezer Yamanel.

**Software:** Erdal Sari.

**Supervision:** Rabia Gönül Sezer Yamanel.

**Visualization:** Erdal Sari.

**Writing – original draft:** Özlem Erdede.

**Writing – review & editing:** Özlem Erdede, Rabia Gönül Sezer Yamanel.
